# Does frequency of resistance training affect tibial cortical bone density in older women? A randomized controlled trial

**DOI:** 10.1007/s00198-012-2000-3

**Published:** 2012-05-12

**Authors:** M. C. Ashe, E. Gorman, K. M. Khan, P. M. Brasher, D. M. L. Cooper, H. A. McKay, T. Liu-Ambrose

**Affiliations:** 1 Center for Hip Health and Mobility, 7F-2635 Laurel Street, Vancouver, BC V5Z 1M9 Canada; 2 Vancouver Coastal Health Research Institute, Vancouver, BC V5Z 3P1 Canada; 3 Department of Family Practice, UBC, Vancouver, BC V6T 1Z3 Canada; 4 School of Human Kinetics, UBC, Vancouver, BC V6T 1Z1 Canada; 5 Centre for Clinical Epidemiology and Evaluation, Vancouver, BC V5Z 1M9 Canada; 6 Department of Anatomy and Cell Biology, University of Saskatchewan, Saskatoon, SK Canada; 7 Department of Orthopaedics, UBC, Vancouver, BC V5Z 1M9 Canada; 8 Department of Physical Therapy, UBC, Vancouver, BC V6T1Z3 Canada; 9 Brain Research Centre, UBC, Vancouver, BC V6T 2B5 Canada

**Keywords:** Aging, Bone density, Bone strength, Resistance training, pQCT

## Abstract

**Summary:**

This randomized controlled trial evaluated the effect of resistance training frequency (0, 1, and 2 times/week) on cortical volumetric bone mineral density (vBMD) at the tibia in older women. There was no mean difference in change in tibial cortical vBMD in older women who engaged in resistance training (RT) one or two times/week compared with the control group over 12 months after adjusting for baseline values.

**Introduction:**

National guidelines recommend RT two to three times/week to optimize bone health. Our objective was to determine the effect of a 12-month intervention of three different RT frequencies on tibial volumetric cortical density (CovBMD) in healthy older women.

**Methods:**

We randomized participants to the following groups: (1) 2×/week balance and tone group (i.e., no resistance beyond body weight, BT), (2) 1×/week RT (RT1), and (3) 2×/week RT (RT2). Treatment allocation was concealed, and measurement team and the bone data analyst were blinded to group allocation. We used peripheral quantitative computed tomography to acquire one 2.3-mm scan at the 50 % tibia, and the primary outcome was CovBMD. Data were collected at baseline, 6 and 12 months, and we used linear mixed modeling to assess the effect at 12 months.

**Results:**

We assessed 147 participants; 100 women provided data at all three points. Baseline unadjusted mean (SD) tibial CovBMD (in milligrams per cubic centimeter) at the 50 % site was 1,077.4 (43.0) (BT), 1,087.8 (42.0) (RT1), and 1,058.7 (60.4) (RT2). At 12 months, there were no statistically significant differences (−0.45 to −0.17 %) between BT and RT groups for mean difference in change in tibial CovBMD for exercise interventions (BT, RT1, RT2) after adjusting for baseline tibial CovBMD.

**Conclusion:**

We note no mean difference in change in tibial CovBMD in older women who engaged in RT one or two times/week compared with the control group over 12 months. It is unknown if RT of 3× or 4×/week would be enough to promote a statistically significant difference in change of bone density.

## Introduction

Maintaining good bone health is an essential part of healthy aging, yet older women have an increased risk of falls and fractures with considerable consequences at both a personal and societal level. Evidence highlights effective lifestyle interventions for healthy bone aging that includes resistance training (RT) [1], walking [2], and a combination of muscle strengthening and walking programs [3]. A meta-analysis by Martyn-St. James and Carroll [2] showed an increase in proximal femur areal bone mineral density (aBMD) as measured by dual-energy X-ray absorptiometry (DXA) in older adults from prescribed walking programs alone. Of note, previous physical activity studies have reported a modest but important 1 % increase at the proximal femur using DXA following RT interventions in postmenopausal women [4, 5]. Despite the evidence supporting physical activity as osteogenic and national guidelines that recommend RT two to three times/week to optimize bone health [6], to our knowledge, the effect of different frequencies of weekly RT on volumetric bone density has not been evaluated in older women.

Resistance training programs are defined by an increased load or force on the target muscle groups. There are a number of modes that are used for RT, including free weights, air pressure systems, and cantilever systems. During the training program, the load is generally progressively increased, as muscle strength is gained. Bone cells (osteocytes) can respond to loads or strain, and over time, bone is thought to adapt its size and shape based upon the forces acting on it, and the greatest force of influence is conferred by the muscle [7]. Animal studies [8] and pediatric research [9] highlight that exercise may potentially exert an influence on bone geometry by increasing periosteal apposition through osteoblast formation [10].

The effect of RT on bone mass in postmenopausal women has most often been evaluated using DXA, where aBMD at the proximal femur was maintained or increased [4, 5, 11–15]. Advanced imaging such as peripheral quantitative computed tomography (pQCT) permits a more comprehensive assessment of the bone, including (1) the ability to separate cortical from trabecular bone compartments, (2) an estimate of volumetric bone mineral density, and (3) a measure of bone strength or resistance to fracture. Most previous studies have examined the effect of twice or three times a week resistance training on bone density [4, 5, 11, 12, 16] based on the American College of Sports Medicine recommended guidelines [6]. Liu-Ambrose and colleagues[17] highlighted an increase in cortical volumetric bone mineral density (CovBMD) at the radius after 6 months of twice per week resistance training in women 75–85 years of age. While other three times per week RT studies in older adults [18, 19] noted significant differences at the distal and midtibia after 12 months, these adaptations were maintained after 1 year following the end of the intervention [20]. Very few studies have compared the effect of different frequencies of RT on bone mass, and to our knowledge, none of them have investigated the effect of RT frequency on CovBMD, total area (ToA), or bone strength. Although current studies provide a general agreement that exercise has bone health benefits, there remains a great opportunity to refine RT for older adults.

Therefore, the primary objective of this analysis was to determine the effect of three different RT frequencies (0, 1, and 2 times per week) on tibial CovBMD in healthy, community-dwelling postmenopausal women aged 65–75 years of age. Our secondary objective was to investigate the effect of RT frequency on ToA and tibial bone strength in older women.

## Methods

The Brain Power Study was a 1-year parallel group randomized controlled trial (RCT) for community-dwelling women aged 65–75 years, and the primary outcome was executive function [21] (Clinical Registration Number: NCT00426881). The present study was an evaluation of the bone health outcomes. We included community-dwelling women aged 65–75 years of age and excluded women who (1) had a history of neurodegenerative disease and/or stroke, (2) were taking psychotropic drugs or antidepressants within the previous 6 months, (3) were taking cholinesterase inhibitors within the previous 12 months, (4) were on estrogen replacement therapy within the previous 12 months, (5) did not speak or understand English, and/or (6) were unable to attend assessments and the intervention at our research center. The local university and hospital ethics review boards approved this study, and all eligible participants gave an informed, written consent prior to participation in the study.

**Fig. 1:**
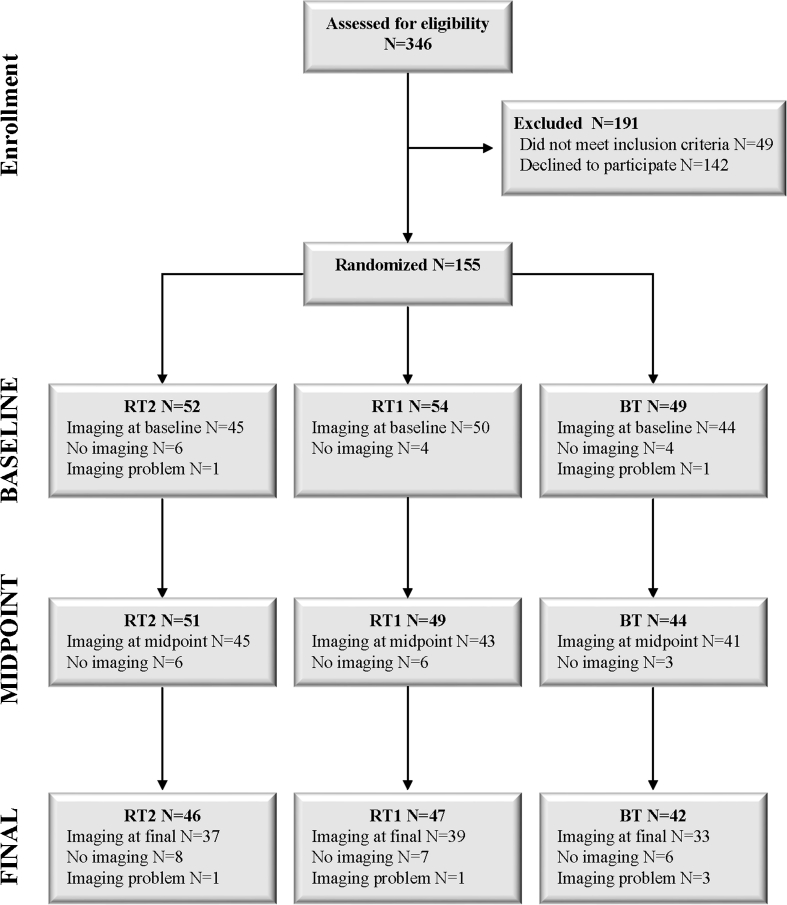
Study flow chart that includes data from the larger trial and the subgroup analysis of bone health outcomes. *BT*, balance and tone; *RT1*, resistance training once per week; *RT2*, resistance training twice per week

We recruited participants through newspaper advertisements, television and radio features, and the provincial physiotherapy professional association. Three hundred and forty-six women were screened and eligible to attend information sessions, after which 155 women were enrolled and assessed. Of the 155 women who were assessed and randomized, 147 women completed the assessment for the bone measures using pQCT at some point during the study (consort flow diagram Fig. 1).

### Randomization: brain power study

After consenting to participate and after baseline assessment was completed, participants were randomly assigned (1:1:1) to one of three groups; we used complete randomization sequencing and a computerized generated system (http://www.randomization.com). The three groups were (1) twice a week balance and tone group (no external resistance other than body weight, BT), (2) once a week resistance training program (RT1), and (3) twice a week resistance training program (RT2). Treatment allocation was concealed, and the measurement team and bone data analyst were blinded to group allocation.

The exercise intervention ran for 1 year (April 2007–April 2008) and was based on the principles of periodization with four terms, each lasting approximately 3 months in duration. Although the intervention was group based, exercises were individualized and the program was progressive so that the exercises in the fourth term built upon the foundation of the previous three terms. All exercise classes were delivered in groups of approximately eight to ten participants, with two certified fitness instructors and one class assistant per class leading each class. All the three groups (BT, RT1, RT2) had similar warm-up and cool-down sessions. The participants in RT1 and RT2 completed eight strengthening exercises for the upper and lower extremities using the Keiser air pressure resistance equipment (Keiser Sports Health Equipment, Fresno, CA) at each session. The participants in RT1 and RT2 completed a one repetition maximum (1RM) at the beginning of each of the four terms, and resistance training was targeted at 8RM; that is, at each session, participants were asked to complete two sets of each exercise at a weight heavy enough that they were able to complete eight repetitions. Every 2 weeks, the exercise instructors increased participants' weights for each exercise if it was appropriate to do so. The BT group completed balance and tone exercises only using the body weight as the resistance. Participants were requested to maintain their usual physical activity routine outside of the classes.

### Sample size

This was an RCT investigating the effect of resistance training on executive function [21]. The size of the trial (52 participants/group) was based on the Stroop test, a measure of selective attention [22], and the trial was designed to have 80 % power to detect differences between groups. During the trial design phase, we also determined if we had adequate power to detect differences between groups for CovBMD; a change prediction of 1 % of tibial cortical density over 1 year for the RT2 group and −1 % for the BT group. Assuming a 20 % attrition rate and using an alpha level = 0.05 (two-sided), we determined that 30 participants per group would provide >80 % power to detect a difference between groups.

### Adverse events

We monitored for any adverse events (e.g., pain, discomfort) at each session; participants were requested to report any events to the instructors who regularly communicated with the research staff.

### Data collection

#### Bone outcomes

We determined CovBMD (in milligrams per cubic meter), ToA (in square millimeter), and tibial bone strength (*I*_max_, in millimeter to the fourth power) using a Norland/Stratec XCT 2000 pQCT (Stratec Medizintechnik GmbH, Pforzheim, Germany) to acquire one 2.3-mm scan at the 50 % site of the left tibia (measured proximally by the length from the lateral malleolus to the knee joint line); the in-plane voxel size was set at 300 μ. Participants were seated comfortably with the left leg supported in position within the scanner. We obtained a scout view and positioned the anatomical reference line at the distal medial edge of the tibia. We reviewed each scan immediately after acquisition and, if movement artifacts were observed, we acquired a second scan. We used customized ImageJ software (NIH, http://rsbweb.nih.gov/ij/) to analyze all scans. Our main outcome was CovBMD (in milligrams per cubic millimeterer) at the middle (50 %) site of the tibia. Our secondary outcomes were ToA (in square millimter) and tibial bone strength (*I*_max,_ in millimmeter to the fourth power). The coefficient of variation (in percent) for the pQCT scanner in our lab for tibial total density and strength strain index was 0.46 and 1.12 %, respectively. All pQCT scans were analyzed by the same trained technician blinded to group allocation.

#### Physical activity

We collected information of the participants' self-reported physical activity in order to determine how much activity occurred outside of the exercise classes. We asked the participants to complete the Physical Activity Scale for the Elderly (PASE), a valid and reliable tool to capture physical activity in the previous 7 days [23]. The PASE consists of ten questions that ask participants to report their physical activity patterns as sedentary, light, moderate, strenuous, strength training, household tasks, and volunteer work. Each section of the questionnaire is weighted according to the effort involved and is reflected in the calculated score.

#### Functional status

We collected information on the participants' functional capacity to engage in physical activity. Participants completed the 6-min walk test (6MWT), a walking test of cardiovascular endurance and functional capacity in older adults [24–26]. We used a 30-m course in a hallway and instructed the participants to walk up and back for 6 min; breaks and mobility aids were permitted and recorded if used. We used standard instructions to the participants, and talking was kept to a minimum. We screened the participants at each time point before undertaking the 6MWT and excluded them if, there was any chest pain, heart attacks, angioplasty, or heart surgery in the previous 3 months, if resting heart rate was above 110 beats per minute, and/or at the discretion of the tester [24].

We assessed the lower extremity strength in sitting using a spring gauge and a padded strap around the tibia; participants were requested to extend the leg. We assessed grip strength with a Jamar handheld dynamometer (JLW Instruments, Sammons Preston, Bolingbrook, IL), using standardized methods. We used the best of three trials for quadriceps muscle and grip strength.

#### Descriptive variables

We collected information on the date of birth and past medical history and medications and asked the participants to complete the Functional Comorbidity Index [27] to ascertain the number of chronic diseases and medications. We measured the height and weight using standard methods, and we calculated the BMI as weight/height^2^ (in kilograms per square meter).

### Statistical analyses

We described the participant characteristics using means and standard deviations or medians and interquartile range if the data were skewed. Participants were analyzed in the exercise group to which they were randomized irrespective of whether they adhered to their intervention. Differences between the proportions of women in each group experiencing an adverse event were analyzed using Pearson's *χ* ^2^ test. Functional status and bone measures (CovBMD, ToA, *I*_max_) were analyzed using linear mixed modeling. The model included exercise group and time as fixed main effects, a group × time interaction and the baseline value of the outcome measure. In addition, random effects for participants were included. We used Stata Software version 11 (StataCorp, TX, USA) for all analyses. All reported *P* values are two sided.

## Results

In the full RCT, 155 women were randomized to one of the three groups and 135 participants completed final assessments for the primary study (87 % compliance). For the analysis of bone outcomes, we assessed the 147 participants and 100 women provided data at all three time points (Fig. 1). The three groups were similar at baseline. Participants were generally active outside of exercise classes and healthy, with few reported chronic health conditions. In addition, 16–21 % of the participants across all the three groups were taking bisphosphonates; the median duration of bisphosphonate use across all the three groups was 48 months or greater. A summary of descriptive variables is provided (Table 1).

**Table 1:** Baseline characteristics of the study participants who underwent imaging analysis of bone health; data are reported as mean (standard deviation), median (interquartile range), or frequency (percent)

Descriptive variables	Balance and tone (*n* = 45)	Once a week (*n* = 53)	Twice a week (*n* = 49)
Age (years)	69.9 (3.1)	69.4 (3.0)	69.2 (3.0)
Height (cm)	161.4 (6.7)	160.8 (7.1)	162.6 (6.6)
Weight (kg)	67.2 (11.4)	68.1 (14.4)	71.2 (14.5)
Body mass index (kg/m^2^)	25.8 (3.8)	26.2 (5.0)	26.9 (4.8)
Number of chronic diseases (*n*)	2 (1–3)	1 (1–2)	2 (1–3.5)
Current bisphosphonate use	9 (20.0 %)	11 (20.8 %)	8 (16.3 %)
Duration of use (median months)	72 (60, 120)	60 (18, 120)	48 (12, 84)
Physical activity			
PASE (median/day)	121.1 (88.5, 156.0)	110.6 (68.3, 147.3)	109.6 (109.6, 162.7) (*n* = 48)
Physical performance			
6MWT (m)	525.9 (72.0) (*n* = 41)	520.1 (62.3) (*n* = 52)	512.2 (95.4) (*n* = 47)
Right grip strength (kg)	22.1 (6.0) (*n* = 44)	21.7 (4.1) (*n* = 51)	21.6 (5.8) (*n* = 48)
Leg strength (kg)	28.2 (7.8)	30.1 (6.7)	29.7 (8.2)

*PASE* Physical Activity Scale for the Elderly

### Exercise class attendance

Exercise class attendance for participants who were imaged using pQCT imaging for BT was 65 %; RT1 was 71 %, and RT2 was 70 %.

### Adverse events

For the full RCT (*n* = 155), 23 women reported adverse musculoskeletal events over the 1-year intervention. There were significant between-group differences (*P* = 0.02) with 5 women from RT2 (*n* = 46, 11 %), 4 women from BT (*n* = 42, 10 %), and 14 women from RT1 (*n* = 47, 30 %) reporting an event. One participant from the BT group had an in-class fall, but no injury was reported. All documented adverse events were resolved within 4 weeks.

### Functional status

Compared with the BT group, the mean difference in change for 6MWT for the RT1 group from baseline to 6 months was 1.6 m (*P* = 0.87) and 11.6 m at 12 months (*P* = 0.40); and for the RT2 group, at 6 months, it was 9.8 m (*P* = 0.34) and 25.0 m (*P* = 0.08) at 12 months.

### Tibial CovBMD

**Fig. 2:**
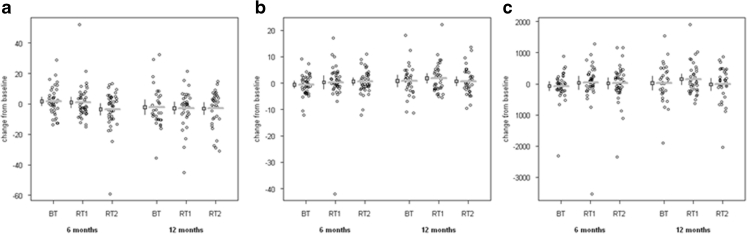
Absolute change from baseline by group (*BT*, balance and tone; *RT1*, resistance training once per week; *RT2*, resistance training twice per week) at the midtibia (50 %) across the three measures of interest. The absolute change from baseline for **a** cortical volumetric bone mineral density (CovBMD, in milligrams per cubic centimeter), **b** total area (ToA, in square millimeter), and **c** bone strength (*I*_max_, in millimeter to the fourth power)

The data are summarized in Table 2, and values at baseline and 6 and 12 months are shown in Fig. 2. After adjusting for baseline tibial CovBMD, there was no statistically significant difference at 12 months between BT and both RT groups, but there was a statistically significant difference between BT and RT2 groups in CovBMD at 6 months. Importantly, all groups maintained tibial CovBMD over 12 months; the estimated mean absolute changes were small (−2.6 (BT), −1.8 (RT1), −4.7 (RT2) mg/cm^3^) representing decreases from the mean baseline score of less than −0.5 %.

**Table 2:** Baseline values with adjusted absolute and percent mean change from baseline by group for tibial cortical volumetric bone density (CovBMD), total area (ToA), and bone strength (*I*_max_) at the midtibia (50 % site) in older women

	Baseline, mean (SD)			6-Month absolute mean change (percent mean change)			12-Month absolute mean change (percent mean change)		
	BT	RT1	RT2	BT	RT1	RT2	BT	RT1	RT2
CovBMD (mg/cm^3^)	1,077.41 (43.1)	1,087.76 (42.0)	1,058.67 (60.4)	2.3 (0.21)	0.84 (0.08)	−4.79 (−0.45)	−2.57 (−0.24)	−1.81 (−0.17)	−4.67 (−0.45)
ToA (mm^2^)	418.12 (51.3)	416.5 (57.72)	426.60 (45.65)	−0.63 (−0.15)	0.61 (0.15)	1.52 (0.36)	1.42 (0.34)	0.86 (0.21)	0.93 (0.22)
*I*_max_ (mm^4^)	19,404.4 (4,515.1)	19,429.93 (5,201.0)	20,169.89 (4,858.2)	−83.26 (−0.43)	69.54 (0.36)	40.82 (0.20)	101.51 (0.52)	124.83 (0.64)	9.94 (0.05)

*CovBMD* volumetric cortical bone mineral density, *I*_*max*_ bone strength, *ToA* total area, *BT* balance and tone, *RT1* resistance training once per week, *RT2* resistance training twice per week

### Tibial area (ToA)

Data are summarized in Table 1, and values at the three time points are shown in Fig. 2. After adjusting for baseline ToA, there were no statistically significant differences between groups at 12 months. The groups maintained total area over 12 months, and the percent change at either 6 or 12 months was ≤0.36 %.

### Tibial bone strength (*I*_max_)

Data are summarized in Table 1, and values at the three time points are shown in Fig. 2. After adjusting for baseline *I*_max_, there were no statistically significant differences between the groups. The groups maintained bone strength over 12 months; the mean difference at either 6 or 12 months, expressed as percent change, was ≤0.65 %.

## Discussion

To our knowledge, this is the first study to investigate cortical bone in response to different frequencies of RT training regimes in postmenopausal women. However, in healthy community-dwelling older women, we note no statistically significant difference between the control group (BT) and the two intervention groups (RT1 and RT2) for tibial CovBMD at 12 months. Although, we did observe a statistically significant difference between BT and RT2 at 6 months, it was less than what has been previously reported as yearly change in CovBMD (−0.5 %) in postmenopausal women [28]; further interpretation of this result must be cautious in view of multiple statistical testing. We also note no statistically significant differences in ToA or tibial bone strength across the three groups at 12 months.

There were no statistically significant differences in CovBMD among exercise groups at 12 months (Table 3), and this is consistent with previous DXA-based studies that have examined the effect of RT on proximal femur aBMD [4, 5, 11, 12] and pQCT studies for this age group [18, 20]. As this is the first study to compare the dose of RT with tibial CovBMD, to our knowledge, it is challenging to compare with previous literature and therefore must rely on previous studies that used different imaging and different study designs. For example, previous literature also highlighted no difference in proximal femur aBMD in premenopausal women [29], postmenopausal women [14], or older men [30] who underwent RT. In addition, although Bemben and colleagues [14] found some positive improvement in hip aBMD, they also observed no significant interactions between groups when they compared different RT frequency (2× vs. 3×/week) and intensity (40 vs. 80 % 1RM). Our results using pQCT to assess bone geometry and the cortical bone compartment specifically extend these studies with similar conclusions.

**Table 3:** Adjusted mean absolute difference for RT1-BT and RT2-BT using linear mixed modeling for tibial cortical volumetric bone density (CovBMD), total area (ToA), and bone strength (*I*_max_) at the midtibia (50 % site) in older women

	RT group mean change − BT mean change, absolute difference (95 % CI)			
	6 Months		12 Months	
	RT1-BT	RT2-BT	RT1-BT	RT2-BT
CovBMD (mg/cm^3^)	−1.46 (−6.28 to 3.36) *P* = 0.55	−7.09 (−11.95 to −2.23) *P* = 0.004	0.76 (−5.32 to 6.85) *P* = 0.81	−2.09 (−8.22 to 4.05) *P* = 0.51
ToA (mm^2^)	1.24 (−1.29 to 3.78) *P* = 0.34	1.49 (−1.05 to 4.04) *P* = 0.25	0.10 (−2.72 to 2.92) *P* = 0.95	−0.49 (−3.34 to 2.35) *P* = 0.73
*I*_max_ (mm^4^)	152.80 (−98.48 to 404.08) *P* = 0.23	124.07 (−129.3 to 377.46) *P* = 0.34	23.32 (−248.86 to 295.5) *P* = 0.87	−91.56 (−366.5 to 183.28) *P* = 0.51

*CovBMD* volumetric cortical bone mineral density, *I*_*max*_ bone strength, *ToA* total area, *BT* balance and tone, *RT1* resistance training once per week, *RT2* resistance training twice per week

There are several plausible explanations as to why there were no differences between groups in cortical bone over 12 months. First, our participants were very active prior to joining the study and outside of the intervention exercise classes over the course of the 12-month intervention. We previously reported [31], using accelerometry in a subset of participants (*n* = 77) from this study, no statistically significant between group differences for moderate to vigorous physical activity (MVPA) outside of the exercise classes and no seasonal differences at four measurement points over the year. Further, for the combined groups, mean MVPA ranged from 24 to 27 min/day depending on the season. It may be that this group of highly motivated participants were already at their “optimum” bone health and had little room for improvement. Although there were increases in the muscle performance measures (one repetition max) in the RT groups over the study [21], there were no statistically significant differences in functional capacity (6MWT) at 6 or 12 months, and this may explain some of the observed statistically nonsignificant differences in bone outcomes.

Frost [32, 33] theorized that older adults might not have the same ability to initiate the bone modeling cycle responsible for changes in cortical bone geometry such as increased total bone area due to periosteal apposition. The Utah paradigm and the strain threshold theory suggest that older adults may not generate enough force or novel strains needed to stimulate bone formation. Thus, the role of physical activity in later life may be to sustain bone strength (by various means) in the aging skeleton [33]. It may also be that bone density is not a sensitive enough measure to assess the effect of RT or physical activity in general [34]. Further, current imaging techniques may not detect small changes in density at the midtibia whereas the distal tibia may be more responsive given its greater amounts of metabolically active trabecular bone.

Exercise acts to stimulate osteoblasts to enhance bone formation, and the first phase includes osteoclastic activity, which removes older bone, followed by the creation of a new hypomineralized tissue. An active remodeling cycle and new bone formation would, in theory, persist throughout an exercise intervention, and this may explain the statistically significant mean difference in CovBMD at 6 months in the RT2 group; although we recognize that the plausible explanation for this finding is due to measurement error. However, in a 2011 Cochrane meta-analysis of exercise and bone health in postmenopausal women, overall, there were positive effects for bone; however, for the combined exercise intervention studies (participants engaged in RT and weight-bearing activities), the authors noted a statistically significant effect favoring the control groups in percent change of aBMD at the hip (−1.07 %, 95 % confidence interval (CI) −1.58 to −0.56) [35].These data highlight the importance of future research to unravel bone response to exercise and physical activity for bone compartments of the aging skeleton.

Our study also raises the question of whether (similar to muscle) there is there an optimum frequency or threshold of resistance exercise that promotes bone strength—after which no further benefit is achieved. In a previous study, once a certain level of muscle strength was reached, once weekly training was sufficient to maintain the benefits [36, 37]. Alternatively, a combination of the RT and exercise outside of the intervention may have sustained cortical density over 12 months in this group of very fit women [3]. The current study cannot provide answers to these questions, and further investigation is required.

### Limitations and strengths

We note that our participants were very active and therefore may not be representative of the general older population and limit the generalizability of the results to a subset of active older women. Second, we acknowledge that pQCT measures bone outcomes at peripheral sites and cannot characterize bone compartments at the clinically relevant proximal femur. Nonetheless, our study includes the novelty of delivering different weekly RT regimens, the length of the exercise intervention, and using pQCT to more aptly assess the cortex.

## Conclusions

Physically active older adult women have the capacity to maintain cortical density, total area, and tibial bone strength over 1 year. The optimal regimen to promote this benefit is not yet clear, and our findings generate hypotheses for future studies that should aim to (1) further investigate the effect of RT frequency on bone geometry and strength, (2) evaluate the effect of RT frequency on less active women, and/or (3) evaluate the effect of combined exercise (walking and RT) on bone strength.
